# Impact of diabetes mellitus on 30-day mortality among acute stroke patients in northern Tanzania

**DOI:** 10.1371/journal.pone.0321988

**Published:** 2025-04-17

**Authors:** Eugénie M. Kamabu, Justin L. Paluku, William P. Howlett, Abid M. Sadiq, Eliada B. Nziku, Doreen T. Eliah, Ibrahim Ali Ibrahim Muhina, Fuad H. Said, Tumaini E. Mirai, Elifuraha W. Mkwizu, Furaha S. Lyamuya, Elichilia R. Shao, Kajiru G. Kilonzo, Venance P. Maro, Sarah J. Urasa, Nyasatu G. Chamba

**Affiliations:** 1 Department of Internal Medicine, Kilimanjaro Christian Medical University College, Moshi, Tanzania; 2 Department of Internal Medicine, HEAL Africa Hospital, Goma, Democratic Republic of Congo; 3 Department of Obstetrics and Gynecology, Faculty of Medicine, University of Goma, Goma, Democratic Republic of the Congo; 4 Department of Internal Medicine, Kilimanjaro Christian Medical Center, Moshi, Tanzania; Radiation Effects Research Foundation, JAPAN

## Abstract

**Background:**

Among acute stroke patients (ASPs), diabetes mellitus (DM) is associated with a higher risk of death, functional dependency, and recurrence. This study aimed to determine the impact of DM on the 30-day mortality among admitted ASPs in northern Tanzania.

**Materials and methods:**

This was a hospital-based prospective cohort study performed among ASPs with and without DM who were admitted to Kilimanjaro Christian Medical Centre from November 2022 to May2023. ASPs were followed for 30 days after the onset of an acute stroke to identify the primary outcome, which was all-cause mortality. Descriptive statistics, logistic regression, and survival analysis were conducted,

**Results:**

Out of 213 ASP, 82 (38.5%) had DM. The overall crude mortality rate was 46.9%. ASPs with DM had a higher mortality rate of 53.7% compared with those without DM (42.7%). A higher proportion of acute stroke patients with DM (84.1%) had a poor outcome (mRS 3-6) (p = 0.038). DM was statistically non-significant for 30-day mortality (aHR 1.56; 95% CI: 0.73–3.32; p = 0.252). However, fever (p = 0.005), severe admission Glasgow coma scale (p = 0.005), severe stroke (p = 0.008), elevated serum creatinine (p = 0.008), and an abnormal respiratory pattern (p = 0.042), were predictors of 30-day mortality,

**Conclusion:**

This study demonstrated a high mortality in ASPs. Although DM did not have a significant impact on 30-day mortality, other factors, such as altered mental state, stroke severity, fever, elevated creatinine, and abnormal respiration, need to be accounted for that may have a significant impact on the mortality in ASPs. These findings highlight the significant burden of DM in stroke patients and underscore the importance of early diagnosis and treatment of ASPs, in the hopes of improving clinical practice and guidelines and reducing morbidity and mortality in Tanzania.

## Introduction

Diabetes mellitus (DM) is a group of metabolic disorders of carbohydrate metabolism in which glucose is both underutilized as an energy source and overproduced due to inappropriate gluconeogenesis and glycogenolysis, resulting in hyperglycemia [1]. Both the incidence and prevalence of DM have been steadily increasing over the past few decades. In 2021, the International Diabetes Federation estimated the prevalence of DM in Tanzania to be 2,884,000 cases (10.3%) among adults [2].

Patients with DM face many complications, including macrovascular complications such as stroke. Historically, stroke was defined as a rapidly developing clinical syndrome of focal or global disturbance of cerebral function lasting more than 24 hours or leading to death, with no apparent cause other than vascular origin. The Stroke Council of the American Heart Association/ American Stroke Association defined ischemic stroke as an episode of neurological dysfunction caused by focal cerebral, spinal, or retinal infarction, which is clinical evidence of an ischemic injury based on neuropathological, neuroimaging, and clinical evidence of permanent injury [3]. A hemorrhagic stroke is a rapidly developing clinical syndrome of neurological dysfunction attributable to a focal collection of blood within the brain parenchyma or ventricular system that is not caused by trauma. Acute stroke is referred to when the patient's presentation at the hospital is ≤7 days after the onset [4]. DM is a well-established risk factor for stroke, and there are several possible mechanisms wherein DM leads to stroke. These include vascular endothelial dysfunction, increased early-age arterial stiffness, systemic inflammation, and thickening of the capillary basal membrane [5]. The reported prevalence of DM among stroke patients ranges between 21% and 44% [6]. Patients with DM have a 1.5–3 times greater risk of stroke, in particular cerebral infarcts, than those without DM [7].

The outcome of patients with stroke can be influenced by a myriad of factors. In sub-Saharan Africa, there is limited healthcare access, delayed hospitalization, a shortage of adequately trained professionals to provide acute care and rehabilitation to stroke patients, as well as a lack of dedicated stroke units [8,9]. This leads to many patients not receiving acute care or adequate care owing to increased morbidity and mortality. In Sub-Saharan Africa, the overall in-hospital mortality rate of acute stroke patients (ASPs) was 22% [10]. The mortality among stroke patients at Kilimanjaro Christian Medical Centre (KCMC) increased from 11.9% in 2017 to 31.8% in 2020 [11,12], and the mortality was higher (61.5%) when ASPs were admitted to the intensive care unit [11].

Additionally, 80% of the causes of mortality within the DM population are linked to cardiovascular disease, with stroke being a major cause [7]. The American Heart Association journal reported that among patients admitted for acute stroke, DM was associated with a higher risk of death, functional dependency, and stroke recurrence [13]. The association between DM and mortality in ASPs at 30 days post-stroke is not well studied in Tanzania, and the impact of DM on stroke outcome is not reported. This study aimed to determine the proportion of DM in stroke patients, the association between DM and stroke outcome, and the independent predictors of mortality in this group of patients at a follow-up of 30 days from admission.

## Materials and methods

### Study design and setting

This was a hospital-based prospective cohort study conducted from 1^st^ November 2022–31^st^ May 2023 at KCMC, Tanzania. KCMC is a national zonal referral hospital located in the urban town of Moshi in the Kilimanjaro region of northern Tanzania. The adult regional population is more than 1,800,000 people, of whom 35% live in urban settings [14]. The Department of Internal Medicine has a total bed capacity of 90 beds and is divided into the medical intensive care unit, high dependency unit, and general ward. The department has subunits, including endoscopy and dialysis units, along with outpatient care units, which have various specialized clinics, including diabetic, cardiac, neurology, and general clinics. Specialized hospital services are limited in sub-Saharan Africa, especially in Tanzania, where it is estimated that there is one practicing neurologist per 8–10 million population [15], with KCMC having at least one computed tomography and one magnetic resonance imaging scanner.

### Ethical considerations

The study obtained Institutional Review Board approval from the Research and Ethics Committee of Kilimanjaro Christian Medical University College (No. PG131/2022). Written consent was obtained from the participants after they were informed of the purpose of the study, either in English or Swahili. When patients were unable to read or write either language, the investigator read and made sure the patients understood the consent, finishing with a signature or thumbprint. In case the patients were unable to consent due to their age (under 18 years), altered mental status, or aphasia, consent was sought from a family member or guardian who was the next of kin. Confidentiality was observed, and all data were stored unlinked to patient identifiers. The participants who did not consent to this study were not denied any form of medical care.

### Data collection

For the sample size calculation of a hospital-based prospective cohort study with a 95% confidence interval and 80% power, estimating a single proportion with p0 = 0.342 [4], this study used the Kish Leslie formula (N=(Zα/2)²p0(1-p0)/(d)²) and calculated a total of 241 patients. With a possible 10% loss to follow-up, the final estimated sample size was 265 patients. The inclusion criteria were patients with an International Classification of Diseases 11th Revision (ICD-11) clinical diagnosis of stroke who presented within 7 days of an acute stroke and were admitted to the medical ward at KCMC. The exclusion criteria included patients with subarachnoid hemorrhage, subdural hematoma, transient ischemic attack, and patients with prior neurologic diseases.

Using a structured questionnaire by the principal investigator, data was collected on socio-demographic characteristics including age, gender, residency, level of education, marital status, insurance status, and employment status. Clinical characteristics included a medical history of DM and hypertension, an abnormal respiration pattern defined as a need for oxygen supplementation and/or an abnormal respiratory auscultation, baseline vital signs, and an admission Glasgow Coma Scale (GCS). The age was categorized based on the WHO’s criteria as young people (18–65 years), middle-aged people (66–79 years), and the aged (80+ years) [16]. Blood pressure readings were recorded according to the 2018 AHA/ACC Hypertension guideline as a standard operating procedure [17]. Hypertension was defined as a blood pressure of ≥140/90 mmHg or a patient on antihypertensive medications [18]. The GCS, based on the level of consciousness post an acute stroke, was categorized as mild (13–15), moderate (9–12), and severe (3–8) [19]. The National Institute of Health Stroke Scale (NIHSS) [20] was used for assessing the stroke severity, which was categorized as follows: minor stroke <4; moderate stroke 5–15; moderate to severe stroke 16–20; and severe stroke >21 [21]. Laboratory investigations included random blood glucose, glycated hemoglobin, serum creatinine, and medical imaging, either a brain computed tomography or brain magnetic resonance imaging. Hyperglycemia was defined according to the American Diabetes Association as random blood sugar >11.1 mmol/L, fasting blood sugar > 7.0 mmol/L or glycated hemoglobin ≥ 6.5% [2]. The primary outcome of interest was all-cause mortality in patients with stroke, and was recorded as ‘dead’ or ‘alive.’ The secondary outcome of interest was the functional outcome, the modified Rankin scale [20], which was used for the assessment among patients with DM and without DM after 30 days post-stroke, according to their level of disability. Through a structured interview with ASPs, questions were asked about their daily activities and functional abilities post-stroke to determine their disability level on a scale from 0 (no symptoms) to 6 (death). This was categorized as a good outcome (0–2) when the patient has no or mild disability and as a poor outcome (3–6) when the patient has severe disability or death.

### Statistical analysis

Data were cleaned, entered, and analyzed using STATA v17. Descriptive statistics were done for all variables. Continuous variables were summarized using the mean (SD) or median (IQR). Categorical variables were summarized using frequency and proportion. The Chi-square test was carried out for each explanatory variable to compare the proportion of DM among stroke patients. Kaplan-Meier analysis was used to describe survival, where the 50th percentile survival time with respective 25% and 75% percentiles was estimated, and significant differences in survival times between DM and non-DM stroke patients were tested using the log-rank test. Crude hazard ratio (cHR) and adjusted hazard ratio (aHR) were analyzed using a Cox proportional hazards model. The process was repeated until the final parsimonious model was obtained without an interaction term, adjusting for confounders, and testing for goodness of fit. The magnitude of the association was interpreted using aHR and 95% confidence intervals (CIs), while a p-value of less than 0.05 was considered to be statistically significant, and those identified based on prior and clinical knowledge of the study setting were included in the adjusted model between stroke and mortality.

## Results

During the study period, a total of 245 patients (18.8% of all admissions) presented with ICD-11 clinical features of stroke. Patients that were excluded from the study were three patients who had a prior history of neurologic diseases, 14 patients who had >7 days of acute stroke onset, seven patients who did not consent, and three patients who were diagnosed with a subarachnoid hemorrhage. Out of 245 patients, 218 (83.5%) met the criteria for acute stroke, among whom 213 (81.6%) completed the 30-day follow-up period and comprised the study cohort. The total number of patients who had DM was 82 (38.5%) out of 213 patients.

The mean ages for stroke patients with and without DM were 66.3±13.6 years and 64.5±17.8 years, respectively. More than half of ASPs were young (<65 years) regardless of those with DM (50%) or without DM (51.1%) (p = 0.756). There was no gender predominance, as the ratio was 1:1 (p = 0.786). A total of 55 (67.1%) patients were married, 59 (72%) had an elementary education level or below, 47 (57.3%) did not have medical insurance, and 44 (53.7%) were unemployed (Table 1).

**Table 1:** Socio-demographic characteristics of acute stroke patients (n=213).

Variables	Total n (%)	With DM n (%)	Without DM n (%)	p value
	213 (100)	82 (38.5)	131 (61.5)	
Age (years) *mean (SD)*	*65.2 (16.3)*	*66.3 (13.6)*	*64.5 (17.8)*	0.756
<65	108 (50.7)	41 (50.0)	67 (51.1)	
66–79	62 (29.1)	26 (31.7)	36 (27.5)	
>80	43 (20.2)	15 (18.3)	28 (21.4)	
Gender				0.786
Male	104 (48.8)	41 (50.0)	63 (48.1)	
Female	109 (51.2)	41 (50.0)	68 (51.9)	
Residence				0.322
Rural	113 (53.1)	40 (48.8)	73 (55.7)	
Urban	100 (47)	42 (51.2)	58 (44.3)	
Marital Status				0.719
Single	15 (7.0)	7 (8.5)	8 (6.1)	
Married	138 (64.8)	55 (67.1)	83 (63.4)	
Divorced	14 (6.6)	4 (4.9)	10 (7.6)	
Widowed	46 (21.6)	16 (19.5)	30 (22.9)	
Education Status				0.645
Illiterate/Elementary	157 (73.7)	59 (72)	98 (74.8)	
High School/College	56 (26.3)	23 (28.0)	33 (25.2)	
Medical Insurance				0.030
Insured	72 (33.8)	35 (42.7)	37 (28.2)	
Non-Insured	141 (66.2)	47 (57.3)	94 (71.8)	
Employment Status				0.205
Employed	25 (11.7)	9 (11)	16 (12.2)	
Unemployed	117 (54.9)	44 (53.7)	73 (55.7)	
Self-Employed	51 (23.9)	17 (20.7)	34 (26)	
Retired	20 (9.4)	12 (14.6)	8 (6.1)	

In this study, the proportion of DM in ASPs was 38.5%, with pre-existing DM in 46 (21.6%) patients, while 36 (16.9%) were newly diagnosed with DM during their hospitalization. Clinical characteristics, as shown in **Table 2**, indicate that the occurrence of ASPs with DM had a history of hypertension (74.4%) compared to those without DM (66.4%) (p = 0.219). Patients in both the DM (76.8%) and non-DM (72.5%) groups had elevated blood pressure on admission. The proportion of patients with a previous history of chronic kidney disease was significantly higher (12.2%) in the DM group than in the non-DM group (4.6%) (p = 0.040). The proportion of patients with fever at the time of admission was significantly higher in ASPs with DM (19.5%) as compared to those without DM (9.9%) (p = 0.047). Ischemic stroke significantly occurred more frequently in the DM group (78.1%) as compared to the non-DM group (57.3%) (p = 0.002).

**Table 2:** Clinical characteristics of acute stroke patients (n=213).

Variables	Total n (%)	With DM n (%)	Without DM n (%)	p value
	213 (100)	82 (38.5)	131 (61.5)	
Previous History of HTN	148 (69.4)	61 (74.4)	87 (66.4)	0.219
HTN on Admission	158 (74.2)	63 (76.8)	95 (72.5)	0.484
Prior History of CKD	16 (7.5)	10 (12.2)	6 (4.6)	0.040
Fever on admission	29 (13.6)	16 (19.5)	13 (9.9)	0.047
Type of Stroke				0.002
Hemorrhagic	74 (34.7)	18 (21.9)	56 (42.7)	
Ischemic	139 (65.3)	64 (78.1)	75 (57.3)	
Elevated Serum Creatinine	77 (36.1)	36 (43.9)	41 (31.3)	0.062
Admission GCS				0.440
Mild	74 (34.7)	31 (37.8)	43 (32.8)	
Moderate	100 (46.9)	34 (41.5)	66 (50.4)	
Severe	39 (18.3)	17 (20.7)	22 (16.8)	
NIHSS				0.759
Minor	39 (18.3)	15 (18.3)	24 (18.3)	
Moderate	79 (37.1)	27 (32.9)	52 (39.7)	
Moderate to Severe	30 (14.1)	13 (15.9)	17 (13.0)	
Severe	65 (30.5)	27 (32.9)	38 (29.0)	
Abnormal respiration	81 (38.0)	34 (41.5)	47 (35.9)	0.414

*CKD: Chronic Kidney Disease; GCS: Glasgow Coma Scale; HTN: Hypertension; NIHSS: National Institutes of Health Stroke Scale*

The present study showed a crude mortality rate of 46.9%. ASPs with DM had a higher mortality rate of 53.7% compared with those without DM (42.7%). However, this difference was not statistically significant (p = 0.121). The proportion of severe disability or death (poor outcome) was significantly higher in DM (85.4%) than in non-DM (71.8%) (p = 0.022), as shown in **Table 3**.

**Table 3:** Association between diabetes mellitus and stroke outcome at 30 days (n=213).

Outcome	Total	With DM	Without DM	p value
	n (%)	n (%)	n (%)	
Modified Rankin Scale				0.022
Good outcome (0–2)	49 (23.0)	12 (14.6)	37 (28.2)	
*Mean (95% CI)*		*1.33 (0.84–1.83)*	*1.32 (1.12–1.53)*	
Poor outcome (3–6)	164 (77.0)	70 (85.4)	94 (71.8)	
*Mean (95% CI)*		*5.17 (4.89–5.45)*	*5.28 (5.07–5.48)*	
Mortality	100 (46.9)	44 (53.7)	56 (42.7)	0.121

**Fig 1:**
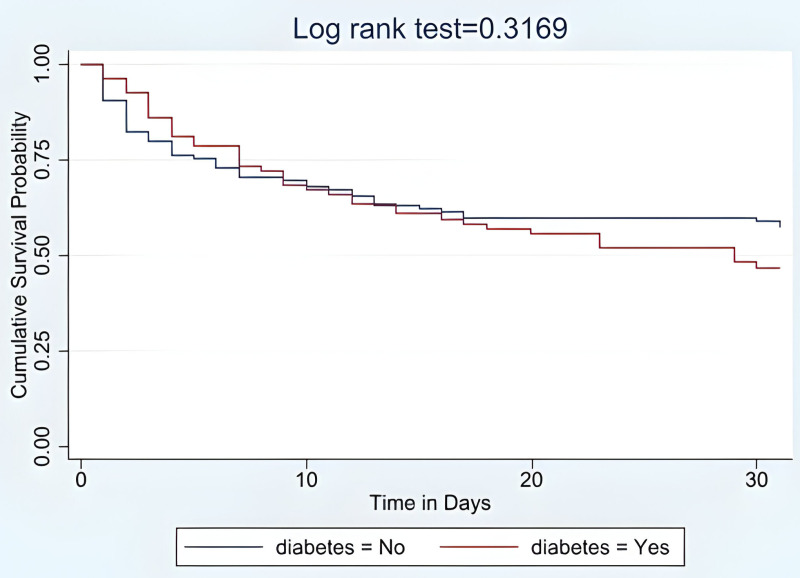
Kaplan-Meier survival rate comparing patients with and without diabetes mellitus after acute stroke.

The median survival time experience was 28 days, with an interquartile range (IQR) of 4–30 days. When comparing the two survival curves of ASPs with DM or without DM seen in **Fig 1**, there was no statistically significant difference between mortality (log-rank test p = 0.3169).

In the crude analysis, DM was statistically non-significant for mortality in 30 days of follow-up (cHR 1.23; 95% CI: 0.81–1.85; p = 0.327). Even after adjusting for other factors, there was no statistical significance (aHR 1.56; 95% CI: 0.73–3.32; p = 0.252). However, in **Table 4**, patients between 66 and 79 years were more likely to experience death (aHR 3.01; 95% CI: 1.07–8.42; p = 0.036). The likelihood of experiencing death was 5.73 times more in ASPs with fever on admission (aHR 5.73; 95% CI: 1.67–19.64; p = 0.005). And 8.78 times greater in those with severe admission GCS (aHR 8.78; 95% CI: 1.88–41.03; p = 0.006). The likelihood of experiencing death was 4.37 times higher in those with severe stroke according to NIHSS on admission (aHR 4.37; 95% CI: 1.37–13.89; p = 0.012). Those with an elevated serum creatinine were 3.18 times more likely to experience death (aHR 3.18; 95% CI: 1.41–7.17; p = 0.005). And 2.08 times elevated in those with abnormality in respiratory pattern (aHR 2.08; 95% CI: 1.08–7.36; p = 0.042).

**Table 4:** Predictors of mortality in acute stroke patients with and without diabetes mellitus (n=213).

Variables	cHR (95% CI)	p value	aHR (95% CI)	p value
Diabetes				
No	1		1	
Yes	1.23 (0.81–1.85)	0.327	1.56 (0.73–3.32)	0.252
*Newly diagnosed*	*1.20 (0.57–2.51)*	*0.632*	*1.35 (0.48–3.80)*	*0.570*
*Pre-existing*	*1.90 (0.96–3.76)*	*0.064*	*1.93 (0.72–5.20)*	*0.191*
Age (years)				
<65	1		1	
66–79	2.93 (1.40–6.13)	0.004	3.01 (1.08–8.42)	0.036
>80	1.99 (0.89–4.44)	0.092	2.89 (0.98–8.55)	0.055
Fever				
No	1		1	
Yes	3.12 (1.92–5.05)	<0.001	5.73 (1.67–19.64)	0.005
Admission GCS				
Mild	1		1	
Moderate	3.23 (1.77–5.86)	<0.001	4.28 (1.07–17.22)	0.040
Severe	11.49 (6.06–21.81)	<0.001	8.78 (1.88–41.03)	0.006
NIHSS on Admission				
Minor	1		1	
Moderate	30.25 (10.01–90.90)	<0.001	11.26 (3.15–40.21)	<0.001
Moderate to Severe	14.25 (6.19–32.82)	<0.001	9.01 (3.26–24.94)	<0.001
Severe	4.21 (1.57–11.29)	0.004	4.37 (1.37–13.89)	0.012
Serum Creatinine				
Normal	1		1	
Elevated	1.73 (1.15–2.59)	0.009	3.18 (1.41–7.17)	0.005
Abnormal Respiration				
No	1		1	
Yes	5.02 (3.27–7.73)	<0.001	2.08 (1.08–7.36)	0.042
Type of Stroke				
Ischemic	1		1	
Hemorrhagic	1.21 (0.69–2.12)	0.515	1.25 (0.53–2.93)	0.605

*GCS: Glasgow Coma Scale; NIHSS: National Institutes of Health Stroke Scale*

## Discussion

In this study, DM was present in 38.5% of patients presenting with acute stroke, over one-third of whom had previously undiagnosed DM. Patients with DM were more likely to have an ischemic stroke. Patients without DM presenting with acute stroke were younger than those with DM. Overall, the 30-day mortality was 46.9%. The presence of DM was not significantly associated with this elevated early mortality. Furthermore, the survival curves of ASPs with and without DM did not show any significant difference in mortality. This insignificant result may be because nearly half of all ASPs died, which is a relatively high percent, regardless of whether the patient had DM or not. Additionally, observing for a longer period may have shown significance. Additionally, this study identified independent predictors of mortality among ASPs, which include fever, severe admission GCS, severe stroke, elevated serum creatinine, and an abnormal respiratory pattern. These factors may have impacted the increased mortality that contributed to the reason why ASPs with or without DM showed no significant difference.

In this study, the proportion of DM among ASPs was high (38.5%) and may be related to lifestyle, culture, dietary habits, and rapid urbanization as seen in Africa, which is associated with the other main cardiovascular risk factors such as hypertension, hyperlipidemia, and obesity. Additionally, similar proportions of DM among ASPs have been reported in some countries in Africa, ranging from 32.2% in Ghana [6] to 34.2% in Cameroon [4]. This similarity could be due to comparable cultures and healthcare challenges. However, previous studies have reported lower frequencies, ranging from 12.8% and 19.7% in Yaoundé in Cameroon [22,23], 18.2% in Dar es Salaam in Tanzania [24], 13.2% in Abidjan in Ivory Coast [7], and 10.7% in Addis Ababa in Ethiopia [25]. These differences could be attributable to differences in study designs, methodologies, and sample sizes or to changing demographic patterns over time. However, healthcare infrastructure and management protocols may differ, resulting in differences in proportions.

This study demonstrates that DM is more likely to be associated with stroke in older people. However, in this cohort, the elderly (>80 years) were less likely to die due to stroke. Similar findings were reported in previous studies [4,6,22] conducted in other African countries. Notably, the findings in this study are different from those reported in India [26] and Copenhagen [27], where stroke was reported to occur at a younger age in persons with DM than in those without DM. This difference may be related to the different ethnicities, geographic regions, and dietary habits, as well as the methodology and sample size. Autonomic dysfunction is known to increase the post-stroke rate of morbidity and mortality [28,29]. Since the sympathetic drive is lower in older patients due to desensitization of the beta-adrenergic receptor with aging, this may explain why older ASPs are less likely to experience death compared to younger patients, as observed in this study [30].

This study revealed that ischemic stroke occurred more frequently in the DM group as compared to the non-DM group. This finding is consistent with other studies reported [6,26,31,32]. Hyperglycemia and insulin resistance observed in DM are known to accelerate the progression of atherosclerosis [33]. Also, nitric oxide-mediated vasodilation is impaired in patients with DM [34]. However, there was no significance in the type of stroke, whether ischemic or hemorrhagic, on the outcomes of ASPs with or without DM.

The current study found that more than one-third of ASPs with DM were unaware of their diagnosis before the stroke event. This may be related to a lack of screening and diagnostic testing for DM in primary care settings or a lack of awareness of the risk of DM in the general population. In Africa, patients typically do not visit healthcare centers for routine medical check-ups and tend to seek care only when they are very ill, which is consistent with previous studies [4,35,36]. Because DM is a well-known risk factor for stroke, this study emphasizes the need to diagnose and treat DM in Tanzania at an early stage before complications emerge.

In this study, the crude mortality rate in ASPs was high (46.9%). Previous studies demonstrate a high mortality rate, such as in Tanzania, where a 30-day post-stroke mortality rate was 37% [20], while in Nigeria, the mortality rate was 30.6% [32]. The findings in this study are in discordance with those reported from Ghana [6] and Ivory Coast [7], where the mortality rate was 17.5% and 26.6% at 30 days post-stroke, respectively. The lower mortality rate reported in those two studies might be attributable to their larger sample size, but also to the fact that patients in those two studies were hospitalized in a specialized stroke unit with appropriate management. These findings underline the necessity for the establishment of dedicated stroke facilities in Africa with a multidisciplinary team.

In this study, ASPs with DM had high 30-day mortality (53.7%) compared to those without DM (42.7%) but was not statistically significant (p = 0.121). In a similar setting, the study found no difference in early mortality between DM (22.1%) and non-DM (20.1%) [22]. This finding was in discordance with other studies done around the world, which found that ASPs with DM had a higher 30-day mortality rate when compared to ASPs without DM [4,37,38]. The DM diagnosis methods, along with different methodologies and the inclusion of a larger sample size, might explain these contradictory results.

Our findings showed that a significant proportion of patients with DM (84.1%) had poor functional outcomes as compared to those without DM (71.8%) (p = 0.038). This means they either died or survived with a severe disability after 30 days of follow-up. Similar findings have been reported in previous studies from around the world that showed poor outcomes in stroke patients with DM compared to those without DM [4,6,26,38,39]. The majority of patients with DM tend to develop neuronal damage due to microvascular complications along with the autonomic dysfunction observed after the stroke event, which might negatively impact their neurological recovery [29].

The present study also analyzed factors that predicted mortality in patients admitted with acute stroke. Previous studies have examined predictors of mortality among ASPs and identified some similar predictors in this study, including elevated body temperature, respiratory disturbances [6], and severe GCS [40]. In addition, stroke severity and elevated serum creatinine were also factors associated with mortality after 30 days of follow-up with ASPs. The severity of renal dysfunction in ASPs may be a marker of end-organ damage due to atherosclerosis and associated risk factors. Although the exact mechanism is not well understood, there is growing evidence that the association between renal dysfunction and ASPs may be a bi-directional causal association, may involve shared risk factors, or be a chance association [41]. Additionally, stroke may influence the central control of the respiratory drive and breathing pattern, airway protection and maintenance, and the respiratory mechanics of inspiration and expiration, which may influence the outcome of ASPs [42]. The determination of predictors of mortality helps identify high-risk patients who may benefit from intensive management strategies that improve the outcome after the stroke event.

The limitations of this study were that it was a single-center hospital-based study, which may not be representative of the burden and characteristics of stroke cases in the whole population. A long-term study analyzing outcomes beyond 30 days may have been beneficial to provide additional data; however, loss of follow-up may affect the results. The presence of multiple comorbidities may affect the 30-day stroke mortality risk, which was not investigated, which may provide another perspective on stroke mortality.

## Conclusion

In conclusion, this study demonstrated a high proportion of DM among ASPs, with more than one-third undiagnosed before admission. Even though the overall crude mortality rate was high in ASPs, DM was statistically non-significant for the elevated 30-day mortality. However, a higher proportion of DM patients experienced poor functional outcomes after the stroke event. Furthermore, severe admission GCS, stroke severity, fever, elevated serum creatinine, and abnormal respiratory patterns were identified as predictors of mortality in ASPs. The findings of this study highlight the significant burden of DM in stroke patients and call for diagnosing and treating DM in Tanzania at an early stage before complications such as stroke emerge. There is a need for dedicated stroke units with multidisciplinary teams in hospitals of developing countries, as the management of ASPs in specialized stroke units showed lower mortality rates compared to those admitted in general wards. This may be established by involving the local government and well-known health organizations in establishing specialized stroke units. And eventually improving the DM patient care and overall healthcare in resource-limited settings.
